# Mesenteric ischemia: the importance of differential diagnosis for the surgeon

**DOI:** 10.1186/1471-2482-13-S2-S51

**Published:** 2013-10-08

**Authors:** Alfonso Reginelli, Francesca Iacobellis, Daniela Berritto, Giuliano Gagliardi, Graziella Di Grezia, Michele Rossi, Paolo Fonio, Roberto Grassi

**Affiliations:** 1 Department of Internal and Experimental Medicine, Magrassi-Lanzara, Institute of Radiology, Second University of Naples, Naples, Italy; 2 Department of Radiology, Azienda Ospedaliera Sant'Andrea, Rome, Italy; 3 Institute of Radiology, University of Turin, Turin, Italy

**Keywords:** Intestinal ischemia, Computed Tomography, Emergency radiology

## Abstract

**Background:**

Intestinal ischemia is an abdominal emergency that accounts for approximately 2% of gastrointestinal illnesses. It represents a complex of diseases caused by impaired blood perfusion to the small and/or large bowel including acute arterial mesenteric ischemia (AAMI), acute venous mesenteric ischemia (AVMI), non occlusive mesenteric ischemia (NOMI), ischemia/reperfusion injury (I/R), ischemic colitis (IC). In this study different study methods (US, CT) will be correlated in the detection of mesenteric ischemia imaging findings due to various etiologies.

**Methods:**

Basing on our institutions experience, 163 cases of mesenteric ischemia/infarction from various cases, investigated with CT and undergone surgical treatment were retrospectively evaluated, in particular trought the following findings: presence/absence of arterial/venous obstruction, bowel wall thickness and enhancement, presence/absence of spastic reflex ileus, hypotonic reflex ileus or paralitic ileus, mural and/or portal/mesenteric pneumatosis, abdominal free fluid, parenchymal ischemia/infarction (liver, kidney, spleen).

**Results:**

To make an early diagnosis useful to ensure a correct therapeutic approach, it is very important to differentiate between occlusive (arterial, venous) and non occlusive causes (NOMI). The typical findings of each forms of mesenteric ischemia are explained in the text.

**Conclusion:**

The radiological findings of mesenteric ischemia have different course in case of different etiology. In venous etiology the progression of damage results faster than arterial even if the symptomatology is less acute; bowel wall thickening is an early finding and easy to detect, simplifying the diagnosis. In arterial etiology the damage progression is slower than in venous ischemia, bowel wall thinning is typical but difficult to recognize so diagnosis may be hard. In the NOMI before/without reperfusion the ischemic damage is similar to AAMI with additional involvement of large bowel parenchymatous organs. In reperfusion after NOMI and after AAMI the CT and surgical findings are similar to those of AVMI, and the injured bowel results quite easy to identify. The prompt recognition of each condition is essential to ensure a successful treatment.

## Background

Several pathologies like cancer, diabetes, neurodegenerative and cardiovascular induced by oxidative stress mechanisms occurs frequently in senescence [1,2]. Oxidant molecules like NO (Nitric oxide) and ROS (Reactive oxygen species) are overproduced in response to drugs, chemicals, high-caloric diets, exercise and other stress agents [3]. Mesenteric ischemia represents an abdominal emergency accounting for approximately 2% of gastrointestinal illnesses [4]. Despite recent advances in surgery and intensive therapy its mortality remains very high, ranging between 50-90% [5]. Patients survival is dependent on prompt recognition and treatment before ischemia progresses to intestinal gangrene [6]. Computed tomography angiography has surpassed angiography as the diagnostic test of choice and represents the gold standard due to its ability to define the arterial anatomy and to evaluate secondary signs of mesenteric ischemia, with sensitivity ranging from 82% to 96% and specificity of 94% [7-12]. Unlike chronic mesenteric ischemia, the treatment of acute mesenteric ischemia remains largely surgical. This is due to the emergent need for revascularization combined with an assessment of bowel viability [7]. An alternative approach might consist in injecting the patients with autologous endothelial progenitor cells, to accelerate tissue revascularization and prevent a surgical approach [13-15]. Unfortunately, the therapeutic feasibility of such a strategy would require the elucidation of the biological properties of these rathe promising cells, which is far from being fully achieved [16-20]. Before proceeding with surgical treatment is necessary to establish the correct etiology as mesenteric ischemia represents a complex of diseases including acute arterial mesenteric ischemia (AAMI), acute venous mesenteric ischemia (AVMI), non occlusive mesenteric ischemia (NOMI) and ischemia/reperfusion injury (I/R). Each of these different forms is characterized by a specific clinical and pathologic time course that must be defined and understood to ensure an appropriate treatment and a good outcome[20,21]. So, aim of this work is to analyze the different clinical pictures of acute mesenteric ischemia related to etiology providing the surgeon with a useful discussion of the differences.

## Materials and methods

Basing on our institutions experience, 163 cases of mesenteric ischemia/infarction from various cases, investigated with CT and undergone surgical treatment were retrospectively evaluated. The following CT findings were considered: patency of the superior mesenteric artery (SMA) or inferior mesenteric artery (IMA), bowel wall thickness (more or less than 3 mm) and enhancement, presence/absence of spastic reflex ileus, hypotonic reflex ileus or paralitic ileus, mural and/or portal/mesenteric pneumatosis, abdominal free fluid, parenchymal ischemia/infarction (liver, kidney, spleen); and the following intraoperative findings: presence of pale/congested mesentery, color of the bowel wall, presence/absence of bowel spasm or dilation, presence/absence of abdominal free fluid, kind of fluid in peritoneal cavity (hemorragic or serous), signs of parenchymal ischemia/infarction (liver, kidney, spleen), extension of the bowel damage. Enhanced CT was performed with 64-detector row configuration (VCT, General Electric Healthcare, Milwaukee, Wis, USA). The following technical parameters were used: in 64-rows CT, effective slice thickness of 3.75 mm for plain acquisition, 1.25 mm in the late arterial phase and 2.5 mm in the portal venous phase; beam pitch of 0.938, reconstruction interval of 0.8 mm, tube voltage of 120-140 KVp and reference mAs of 250/700 mA. Automatic tube current modulation was used to minimize the radiation exposure. A standard reconstruction algorithm was used. Patients were instructed not to breath during helical imaging to avoid motion artefacts. All patients received iodinated nonionic contrast material (iopromide, Ultravist 300, Schering, Berlin, Germany) intravenously at a rate of 3.5 mL/s with a power injector. No patient received oral contrast material.

## Results and discussion

Patients typically present in their 60s to 70s and often have a number of medical comorbidities. In case of occlusive etiology, abdominal pain is the most common presenting symptom (94%) and patients usually complain of abdominal pain out of proportion to the abdominal examination. Other symptoms include nausea (56%), vomiting (38%), diarrhea (31%), and tachycardia (31%). In advanced phase, the patient develops peritoneal signs of distention, guarding, rigidity, and hypotension[22-25]. NOMI is suggested by medical history of systemic hypoperfusion due to major surgery, cardiac impairment, hemorrhage, shock, cirrhosis, sepsis, chronic renal disease, medications, and the use of splanchnic vasoconstrictors [26].

### Acute arterial mesenteric ischemia

The diagnosis of AMI can be difficult, because most patients have nonspecific symptoms of abdominal pain. Abdominal pain out of proportion to the findings on physical examination and persisting beyond 2 to 3 hours is the classic pre- sentation [26]. At least the 65% of cases of intestinal ischemia are due to arterial embolism or thrombosis with blood flow impairment in the superior mesenteric artery (SMA) distribution affecting all or portions of the small bowel and right colon [26]. Most embolic events are thromboembolic in nature and arise from a cardiac source. Risk for a thromboembolic event include atrial tachyarrythmias, low ejection fraction (congestive heart failure, cardiomyopathy), recent myocardial ischemia or infarction, and ventricular aneurysms [7].

Thanks to the development of animal model of mesenteric ischemia, it was possible to explore the physiopathological process that conducts to intestinal infarction, underlining that a specific timing of lesions occurs and it is very important to define this to ensure a correct diagnostic assessment and therapeutic result [27].

Enhanced CT findings are conditioned by the involved tract (some intestinal segments are more sensitive to ischemic injury) by the typology (varying according to the obstructive mechanisms) and by the timing.

**Figure 1:**
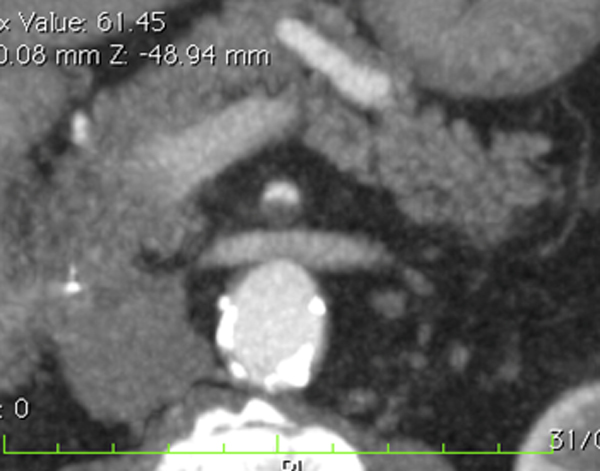
**Acute arterial mesenteric ischemia Contrast-enhanced MDCT 2D reconstruction on axial plane shows thrombosis with impairment in the blood flow in the superior mesenteric artery (SMA)**.

In the early phase the presence of emboli or thrombi as filling defect in the lumen of the artery could be found (Figure 1). Their identification can be difficult if they are small and peripherally localized. The injured small bowel loops are contracted in consequence of spastic reflex ileus and intestinal wall shows lacking of/poor enhancement. The mesentery is bloodless, due to reduction in vessels caliber [4,8,11].

Hyperperistalsis and hyperactive bowel sounds on auscultation are evident. In case of laparotomy the bowel appear spastic in response to the ischemic damage, with pale mesentery[26].

**Figure 2:**
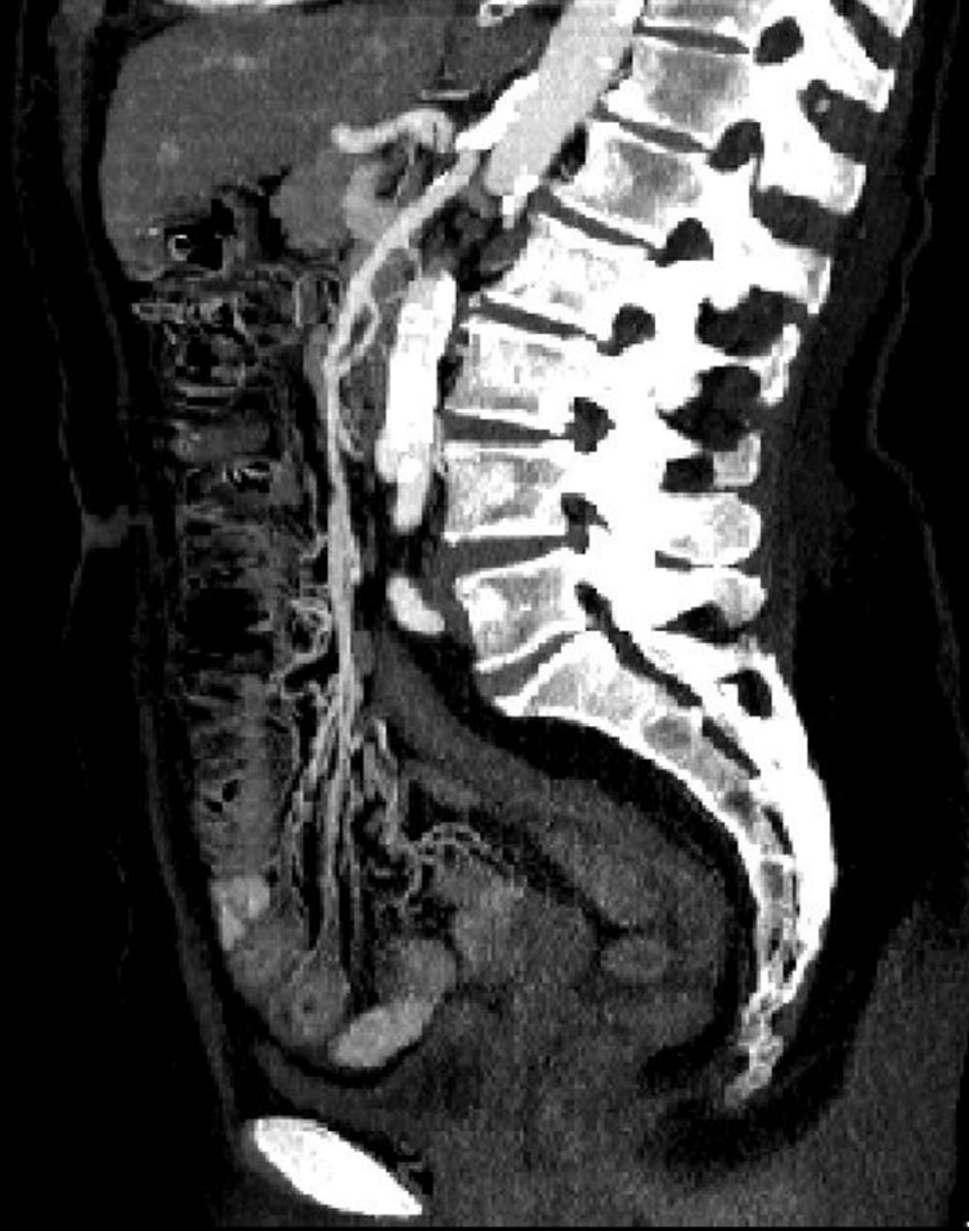
**Acute arterial mesenteric ischemia**. Contrast-enhanced MDCT 2D reconstruction on sagittal plane in early phase: the CT shows the presence of emboli or thrombi as filling defect in the lumen of the artery. If they are small and peripherally localized, the identification can be difficult. The loops of injured small bowel are contracted in consequence of spastic reflex ileus and intestinal wall shows lacking of/poor enhancement. The mesentery is bloodless, due to reduction in caliber of the vessels and apparently in number.

In the intermediate phase, blood and fluids are drained by the venous system, and the bowel wall become thin, with a typical "paper thin" aspect [27,28]; the intestine from the spastic reflex ileus evolves into hypotonic ileus and the loops appear only gas filled, also peritoneal free fluid can be detected [29] (Figure 2). At laparotomy it is possible to find a small amount of serous free fluid in peritoneal cavity, some loops may appear more injured than others with initial change in color from pink to the brownish; the loops appear distended too. In this stage, the pain may be diminishing but becomes more continuous and diffuse. The abdomen becomes distended, and there is more generalized tenderness. Bowel sounds are absent. If the causative factor is not removed, the ischemia rapidly evolves into infarction and the patient clinical picture worsens with fluid, electrolyte and pH alterations. Air-fluid levels appear as progression from hypotonic reflex ileus in paralytic ileus [11]. Unfortunately, many patients are diagnosed in this stage because they are overlooked or not identified in previous phases. The wall necrosis lead to parietal, mesenteric, and even portal pneumatosis [29] or perforation with pneumo-peritoneum, retro-pneumo-peritoneum; the presence of free fluid in the abdominal cavity [30-33] is due to increased hydrostatic pressure inside the intestinal loops that allows extravasation of plasma and to the peritoneal reaction to the ischemic injury. At laparotomy serous or hemorragic free fluid is found, the intestine appears charcoal black due to necrosis, and the loops are dilated. If the extent of damage is still limited, a bowel resection can be performed, even if in the majority of cases the damage is so extensive that the prognosis is poor [34,35].

### Acute venous mesenteric ischemia

AVMI account for 10% of cases of intestinal ischemia [36]. Impaired venous drainage disease presents a less acute symptomatology than arterial ischemia, even if both macroscopical and CT findings are more evident and striking if compared with arterial etiology[11,36]. The impairment in the intestinal vein drainage due to SMV occlusion causes vascular engorgement, swelling, and hemorrhage of the bowel wall, with extravasation of fluid to the peritoneal cavity; also mucosal edema and punctate hemorrhage that progress to widespread hemorrhages may be found at surgical exploration. Progression of the thrombosis and inadequate collateral circulation leads to infarction of the jejunum and the ileum [37].

**Figure 3:**
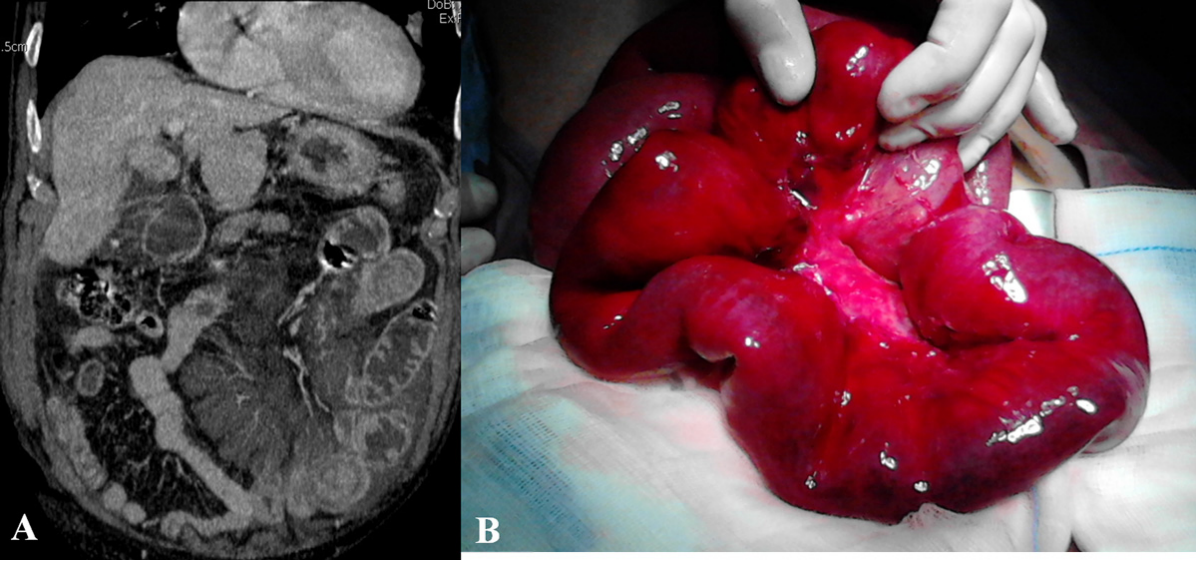
**Acute venous mesenteric ischemia Contrast-enhanced MDCT 2D reconstruction on coronal plane in cases of mesenteric venous thrombosis (a) confirmed at surgery (b)**.

At CT the thrombus may be seen in the SMV [26], in early phase the main findings are represented by mural thickening, intramural hemorrhage, and submucosal edema. The target appearance of the ischemic bowel at CT consists in an inner hyperdense ring due to mucosal hypervascularity, hemorrhage, and ulceration, a middle hypodense edematous submucosa and a normal or slightly thickened muscularis propria. In this phase the intestine and the mesentery are dark red colored and congested, free fluid is present (Figure 3a-b). If the vascular impairment persists there is an increase of hemorragic free fluid, bowel wall becomes necrotic and peritonitis develop. So the main CT findings are: mural thickening of the involved segments, peritoneal fluid, and mesenteric engorgement. In late stage venous thrombosis, absence of mural enhancement, and the presence of fluid and gas may be evident in the mesenteric and portal veins, bowel wall, and sub-peritoneal or peritoneal space. At surgical exploration the injured loops appear manifestly necrotic. A resection may not be enough to save the patient if the damage is very extensive [11,26,38].

### NOMI

**Figure 4:**
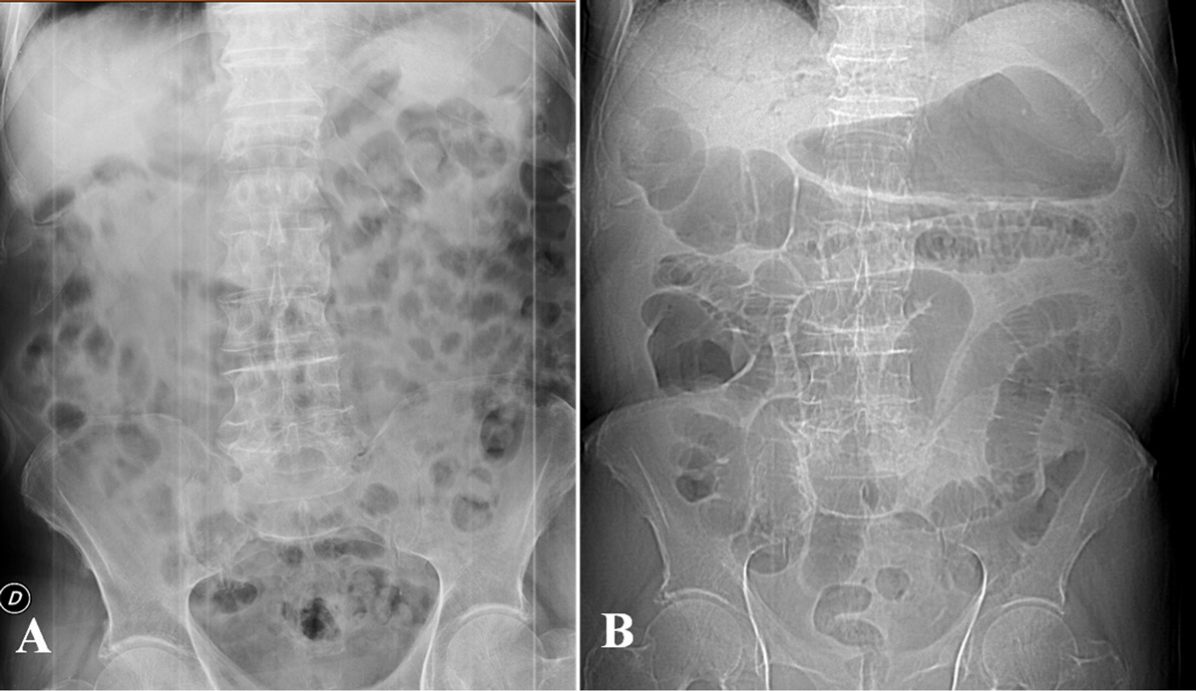
**NOMI**. Plain abdominal film shows in early phase: ischemia due to vasoconstriction of the splanchnic vessels leading to spastic reflex ileus(a) and in intermediate phase: the bowel wall of both small and large bowel appear thinned (b).

NOMI represents the 20-30% of all cases of acute mesenteric ischemia [39-43] and comprises the forms of mesenteric ischemia with no occlusion of the mesenteric artery or vein in the area of bowel necrosis [44,45]. NOMI is commonly caused by decreased cardiac output resulting in splanchnic hypoperfusion. It generally affects patients over 50 years of age suffering from myocardial infarction, congestive heart failure, aortic insufficiency, and renal or hepatic diseases [39,40,45]. During low flow states, the entire intestine can be damaged, but the small intestine and the right colon seem to be more sensitive to the states of shock [46-48]. Since the reduction in blood flow affects both the SMA and IMA, all collateral circulations are ineffective. The ischemic lesions and imaging findings have a similar evolution in the small and in the large intestine. In the small intestine, the CT and surgical findings are the same of AAMI if the blood pressure is not restored. In the early phase the main finding is represented by the spastic reflex ileus due to semilunar fold contraction in response to the ischemia, mesenteric vasoconstriction may be seen as early as 10 min after the onset of hypotension [39] (Figure 4a-b). At enhanced CT, the mesenteric vessels appear patent and the intestinal wall shows a reduction of the enhancement [11]. Surgical intervention could be required if the ischemic damage progresses. In the intermediate phase the bowel wall of both small and large bowel appear thinned [44] due to inefficacy to collateral circulation. All loops are dilated only gas filled and the transition from spastic ileus to hypotonic ileus is detected. The mesentery appear pale and there is also lack of enhancement of the intestinal wall [11,29,39]. If the blood pressure is restored, the intestine is reperfused and the findings depends on the severity of the ischemic damage. The edema of the wall cause low attenuation to enhanced CT and the typical "target sign". A normal enhancement of the intestinal mucosa is a sign of bowel life [49-51]. In the late phase prolonged ischemia, ineffective reperfusion or reperfusion injury, however, can lead to trans-mural necrosis. The intestinal segments appear dilated and distended by air-fluid levels, resulting in paralytic ileus. The absence of enhancement is a sign of ineffective reperfusion which suggests the need for a surgical resection. In the NOMI, due to its pathogenesis, there is also an ischemic involvement of parenchymatous organs (liver, spleen, kidney) that should be carefully investigated [52].

### Ischemia/Reperfusion injury

Ischemia-reperfusion injury (I/R) of the intestine is an important factor associated with a high morbidity and mortality [53]. It may represent the consequence of revascularization after AAMI or of restored blood pressure after low flow states (NOMI). The initial damage caused by ischemia is further worsened by reperfusion [53]; paradoxically, restoration of blood flow to the ischemic tissue initiates a cascade of events that may lead to additional cell injury known as reperfusion injury due to the development of reactive oxygen species. This reperfusion damage frequently exceeds the original ischemic insult [54]. Consequently, many cases of intestinal I/R develop into shock, multiple organ failure, and death [22,27,55,56]. Distinguish between mesenteric ischemia with and without reperfusion have a great clinical importance because these conditions have different therapeutic approaches [57,58]. When reperfusion occurs, the reperfused intestine may have a different pattern [59-65]; due to the microcirculation injury there is extravasation of plasma and red blood cells with hemorrhagic foci, the CT and surgical findings are similar to those of AVMI with bowel wall thickening, target sign, free hemorragic fluid and change in color of the bowel wall. The entity and extension of damage are related with the duration and degree of ischemia; they may progress to the necrosis of the entire wall if reperfusion is ineffective or in restored bowel wall condition and free fluid resorption in case of timely and effective reperfusion[26].

## Conclusions

The radiological findings of mesenteric ischemia have different course in case of different etiology. In venous etiology the progression of damage results faster than arterial even if the symptomatology is less acute; bowel wall thickening is an early finding and easy to detect, simplifying the diagnosis. In arterial etiology the damage progression is slower than in venous ischemia, bowel wall thinning is typical but difficult to recognize so diagnosis may be hard. In the NOMI before/without reperfusion the ischemic damage is similar to AAMI with additional involvement of large bowel parenchymatous organs. In reperfusion after NOMI and after AAMI the CT and surgical findings are similar to those of AVMI, and the injured bowel results quite easy to identify. The prompt recognition of each condition is essential to ensure a successful treatment.

## Competing interests

The authors declare that they have no competing interests.

## Authors' contributions

FI: conceived the study, carried out the examinations, analyzed and interpreted the data, drafted the manuscript.

AR: conceived the study, carried out the examinations, analyzed and interpreted the data.

DB: conceived the study, carried out the examinations, analyzed and interpreted the data.

GG: critically revised the manuscript.

GDG: critically revised the manuscript.

MR: critically revised the manuscript.

PF: conceived the study and critically revised the manuscript.

RG: conceived the study, analyzed and interpreted the data, critically revised the drafted manuscript.

All authors read and approved the final manuscript.

## Authors' information

FI: Resident in Radiology Training Program at Second University of Naples

AR: Post-Doctoral Fellow in Radiology at Second University of Naples

DB: Resident in Radiology Training Program at Second University of Naples

GG: Resident in Radiology Training Program at Second University of Naples

GDG: Resident in Radiology Training Program at Second University of Naples

MR: Assistant Professor of Radiology, University of Rome "Sapienza"

PF: Associate Professor of Radiology at University of Turin

RG: Full Professor of Radiology, Second University of Naples
